# Role of ^18^F-FDG PET/CT in Head and Neck Squamous Cell Carcinoma: Current Evidence and Innovative Applications

**DOI:** 10.3390/cancers16101905

**Published:** 2024-05-16

**Authors:** Carmelo Caldarella, Marina De Risi, Mariangela Massaccesi, Francesco Miccichè, Francesco Bussu, Jacopo Galli, Vittoria Rufini, Lucia Leccisotti

**Affiliations:** 1 Nuclear Medicine Unit, Department of Radiology and Oncologic Radiotherapy, Fondazione Policlinico Universitario A. Gemelli IRCCS, 00168 Rome, Italy; carmelo.caldarella@policlinicogemelli.it (C.C.); marina.derisi@guest.policlinicogemelli.it (M.D.R.); lucia.leccisotti@unicatt.it (L.L.); 2 Radiation Oncology Unit, Department of Radiology and Oncologic Radiotherapy, Fondazione Policlinico Universitario A. Gemelli IRCCS, 00168 Rome, Italy; mariangela.massaccesi@policlinicogemelli.it; 3 Radiation Oncology Unit, Ospedale Isola Tiberina—Gemelli Isola, 00186 Rome, Italy; francesco.micciche@fbf-isola.it; 4 Otorhinolaryngology Operative Unit, Azienda Ospedaliero Universitaria Sassari, 07100 Sassari, Italy; fbussu@uniss.it; 5 Department of Medicine, Surgery and Pharmacy, University of Sassari, 07100 Sassari, Italy; 6 Otorhinolaryngology Unit, Department of Neurosciences, Sensory Organs and Thorax, Fondazione Policlinico Universitario A. Gemelli IRCCS, 00168 Rome, Italy; jacopo.galli@unicatt.it; 7 Section of Otolaryngology, Department of Head-Neck and Sensory Organs, Università Cattolica del Sacro Cuore, 00168 Rome, Italy; 8 Section of Nuclear Medicine, Department of Radiological Sciences and Hematology, Università Cattolica del Sacro Cuore, 00168 Rome, Italy

**Keywords:** head and neck, squamous cell carcinoma, ^18^F-FDG, PET, PET/CT, PET/MRI, radiomics

## Abstract

**Simple Summary:**

Among head–neck tumors, squamous cell carcinoma is the most frequent histotype and includes a range of malignancies with different sites of origin as well as different therapeutic strategies and clinical outcomes. In daily practice, patients with head–neck squamous cell carcinoma are seen in various clinical settings, requiring a multidisciplinary approach to therapeutic decisions and clinical care. ^18^F-FDG PET/CT plays a well-defined role in the management of these tumors for pre-treatment staging and radiotherapy planning as well as treatment-response assessment and post-therapy follow-up. This paper is an overview of the standard use of ^18^F-FDG PET/CT in the various clinical scenarios of head–neck squamous cell carcinoma. Also, emerging applications will be reviewed, including the use of radiopharmaceuticals other than ^18^F-FDG, PET/MRI implementation in clinical practice, and the use of radiomics and machine learning.

**Abstract:**

This article provides an overview of the use of ^18^F-FDG PET/CT in various clinical scenarios of head–neck squamous cell carcinoma, ranging from initial staging to treatment-response assessment, and post-therapy follow-up, with a focus on the current evidence, debated issues, and innovative applications. Methodological aspects and the most frequent pitfalls in head–neck imaging interpretation are described. In the initial work-up, ^18^F-FDG PET/CT is recommended in patients with metastatic cervical lymphadenectomy and occult primary tumor; moreover, it is a well-established imaging tool for detecting cervical nodal involvement, distant metastases, and synchronous primary tumors. Various ^18^F-FDG pre-treatment parameters show prognostic value in terms of disease progression and overall survival. In this scenario, an emerging role is played by radiomics and machine learning. For radiation-treatment planning, ^18^F-FDG PET/CT provides an accurate delineation of target volumes and treatment adaptation. Due to its high negative predictive value, ^18^F-FDG PET/CT, performed at least 12 weeks after the completion of chemoradiotherapy, can prevent unnecessary neck dissections. In addition to radiomics and machine learning, emerging applications include PET/MRI, which combines the high soft-tissue contrast of MRI with the metabolic information of PET, and the use of PET radiopharmaceuticals other than ^18^F-FDG, which can answer specific clinical needs.

## 1. Introduction

Head–neck (HN) cancer, which is the seventh most common malignancy worldwide [1] refers to a heterogeneous group of tumors. Squamous cell carcinoma (SCC) is the most frequent histotype (up to 90% of all HN cancers), including a range of malignancies with different sites of origin as well as different therapeutic strategies and clinical outcomes [2,3]. Less common types of HN tumors are lymphomas, thyroid, or salivary gland tumors, paragangliomas, and skin cancers, each with a specific clinical behavior and treatment [4,5].

Different imaging techniques are available with specific distinctive features that make their use complementary in the diagnostic work-up of patients with HN tumors. Ultrasonography is useful to detect cervical lymph nodes (LNs) and guide fine needle aspiration [6]. Due to the high spatial resolution and tissue contrast, contrast-enhanced computed tomography (ceCT) and magnetic resonance imaging (MRI) are the techniques of choice for primary tumor evaluation and loco-regional staging, with preferential indications for each of them (for example, MRI is preferred over CT to assess skull-base invasion and perineural spread) [2,7]. However, their role in detecting lymph nodes and distant metastases is suboptimal, except for lung metastases that are better detected by chest CT [2,8,9]. ^18^F-fluorodeoxy-D-glucose positron emission tomography/computed tomography (^18^F-FDG PET/CT) is a well-established imaging tool in the management of HNSCC. The National Comprehensive Cancer Network (NCCN) guidelines support the use of ^18^F-FDG PET/CT in patients with advanced-stage disease in different clinical settings [2,3,10,11,12,13]. Various clinical scenarios confirm the versatility and importance of ^18^F-FDG PET/CT for the initial work-up (treatment planning) and treatment-response assessment, as well as for prognostic evaluation of HNSCC [10,11,12,14,15]. Among these is its ability to identify the occult primary tumor in cases of metastatic cervical involvement, thus guiding biopsy on the suspected site and allowing direct radiotherapy on the selected target only with relevant therapeutic advantages [16,17]. Moreover, due to its high negative predictive value, ^18^F-FDG PET/CT performed 3 months after the completion of chemoradiotherapy, can prevent unnecessary neck dissections, with fewer complications and adverse effects, an approach that is cost effective [14,18]. Radiopharmaceuticals other than ^18^F-FDG are available; they target specific biological features of HN tumors, such as hypoxia, protein synthesis, cell proliferation, somatostatin receptor expression, and others, with a potential added value in specific clinical settings [19,20,21,22,23,24,25].

In recent years, PET/CT scanners, which allow the correlation of anatomical with functional information, have gained important technological innovations resulting from advances in detector hardware, such as digital systems, and improvements in image reconstruction algorithms, such as time of flight, providing quick and high-resolution imaging with increased image quality [26,27,28,29]. Most recently, long-axial field of view (LAFOV) PET/CT systems allow for the simultaneous scanning of a large portion of the body (between 106 and 194 cm, according to the PET/CT scanner), with higher physical sensitivity and spatial resolution and reduced acquisition time than standard PET/CT devices [30,31,32,33,34]. Moreover, since the last decade, the integrated functional–anatomic imaging of HN cancers has been taking advantage of the development of PET/MRI technology, which combines the high soft-tissue contrast of MRI with the metabolic information on a patient’s disease status of PET [35]. Finally, the application of radiomics and machine learning is gaining more and more interest in oncologic imaging, including HN cancer imaging [36,37]. This article provides a comprehensive overview of the standard use of ^18^F-FDG PET/CT in the various clinical scenarios of HNSCC. Other topics that deserve to be addressed are the use of PET radiopharmaceuticals other than ^18^F-FDG in specific clinical settings of HNSCC or different histotypes, as well as PET/MRI implementation in clinical practice and the new perspectives offered by radiomics and machine learning.

## 2. Methodological Aspects and Pitfalls in Imaging Interpretation

Patient preparation and image acquisition are essential to optimize PET/CT imaging and interpretation and are performed according to international guidelines [38]. Patients are advised to fast 4–6 h prior to tracer injection to avoid an increase in glucose levels and the consequent reduction of ^18^F-FDG uptake by tumor cells due to the saturation of glucose transporters on the cell membrane [4]. For routine clinical studies, a plasma glucose level lower than 200 mg/dL is recommended [38]. When blood glucose levels are >200 mg/dL, the administration of rapid-acting insulin may be considered. However, an interval of ≥4 h between insulin injection and ^18^F-FDG administration must be respected to avoid the increased ^18^F-FDG muscle uptake induced by insulin [38]. For this reason, in clinical routine, this solution is rarely feasible. In our unit, we have adopted the following operative procedure:
**Blood Sampling****Serum Glucose Level****Prescription**Basal≤200 mg/dL^18^F-FDG injection
200–300 mg/dLInvite the patient to hydrate and walk for at least 30 min and recheck serum glucose levels
>300 mg/dLRescheduleAfter hydration and walking≤200 mg/dL^18^F-FDG injection
↓ but still >200 mg/dLDecision of rescheduling or injecting ^18^F-FDG made by nuclear medicine physician 
Further ↑Reschedule↓ : decrease; ↑ : increase.

**Figure 1:**
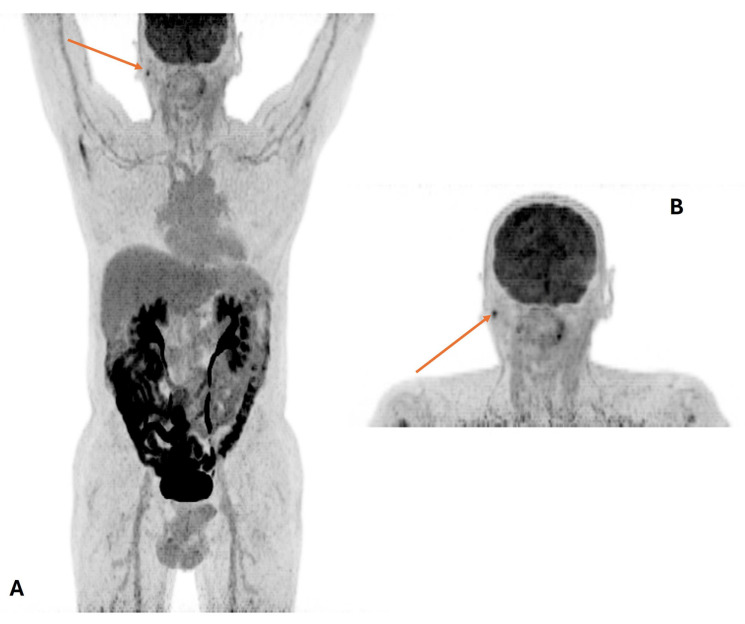
Example of recommended PET acquisition protocol for head-and-neck tumors. Skull base to proximal thighs scan with arms raised (**A**) followed by head–neck study with the patient’s arm placed down (**B**). The focal uptake in the right parotid gland (arrow) is a reactive lymph node.

After the ^18^F-FDG injection, patients are invited to rest and stay calm to minimize skeletal muscle uptake. To avoid brown fat activation in the HN region, a warm temperature in the injection room and a blanket put to cover the neck and thorax are advised [4]. Standard PET/CT imaging extends from the skull base to the upper thighs and is usually performed by acquiring low-dose unenhanced CT, providing high diagnostic performance for tumor staging. In selected cases, the use of intravenous contrast media may add diagnostic information [39,40]. To reduce artifacts on the CT (beam-hardening artifacts) or those due to attenuation correction on PET images in the HN region, the patient’s arms should be placed down. The use of dedicated acquisition protocols (e.g., a standard imaging from the skull base to mid thighs with the patient’s arms above the head followed by a PET/CT study of the neck with the patient’s arm placed down), as well as reducing artifacts, may improve the detectability of small lymph nodes [41,42,43] (Figure 1). PET/CT requires patient cooperation. To avoid misregistration artifacts and pitfalls in the interpretation of fused images, HN positioning as well as lack of motion of the HN during image acquisition are of critical importance. In selected cases, e.g., when the PET/CT study is performed for radiation planning, a neck immobilization device should be used [28]. 

In addition to image artifacts, PET/CT image interpretation may be hampered by the complex anatomy and the small size of the anatomical structures of the HN region, as well as the physiological and widely variable uptake of ^18^F-FDG in normal tissues, including vocal cords, salivary glands, cervical muscles, lymphoid tissue, and brown fat [43,44]. Various benign tumors, such as Warthin’s tumor, show increased ^18^F-FDG uptake [45]. Also, inflammatory processes can cause false-positive results due to ^18^F-FDG uptake by activated inflammatory cells, particularly those occurring in patients submitted to biopsy, surgery, or radiotherapy. Additionally, the presence of high-density material, such as metal dental prostheses or a chemotherapy port, or the presence of calcified lymph nodes, may further compromise the interpretation of PET/CT images, thus requiring non-attenuation corrected PET data evaluation. Also, standardized uptake value (SUV) measurements may be impaired [44]. A potential pitfall in image interpretation is the presence of perineural spread of HN cancer, as nuclear medicine physicians could be not familiar with this finding, which is usually characterized by a linear or curvilinear FDG uptake along the distribution of a certain cranial nerve [46]. Finally, knowledge of the patient’s medical history, including oncological history and relevant comorbidities (especially infection/inflammation), as well as the results of other imaging tests, is of utmost importance for the appropriate interpretation of PET/CT images. All relevant findings at PET/CT should be reviewed and discussed by a multidisciplinary team [1,38].

## 3. Pre-Treatment Staging

For HN tumors, the diagnostic process usually starts with a clinical examination by the specialist physician followed by an endoscopy and biopsies to assess the correct histological diagnosis. The next step is the accurate staging of the disease, which is necessary to reach the most appropriate treatment plan and obtain prognostic information.

### 3.1. Primary Tumor Assessment

**Figure 2:**
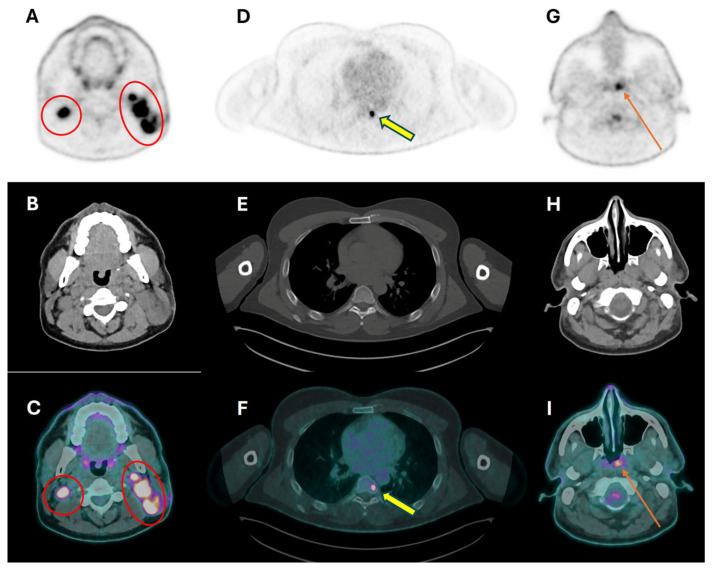
Axial PET, CT, and PET/CT images of a patient with cervical lymph node metastases of undifferentiated carcinoma, and no clinical evidence of primary lesion in the head–neck district. High ^18^F-FDG uptake is seen in enlarged cervical lymph nodes bilaterally (**A**,**B**), although more evident on the left side (red circles in (**A**,**C**)), and in the 7th dorsal vertebra (yellow arrow in (**D**,**F**)) without structural alterations at low-dose CT (**E**). PET/CT scan revealed the otherwise unknown primary nasopharyngeal tumor, on the left side (long arrow in (**G**,**I**)), not apparent on low-dose CT (**H**).

Even though ^18^F-FDG PET/CT is highly sensitive in detecting primary HNSCC (>95% sensitivity), primary tumor assessment is usually performed with CT and/or MRI [47]. The main limitation of PET/CT, especially when performed with low-dose unenhanced CT, is its low spatial resolution, which does not allow for assessing tumor extension, as well as invasion, of adjacent structures. Conversely, ceCT and MRI have higher spatial resolution and tissue contrast, with preferred locations for one or another of these modalities [47]. Indeed, MRI is the preferred imaging modality in nasopharynx, oropharynx, and oral cavity tumors, as well as for perineural spread and bone marrow involvement, while ceCT is preferred for larynx tumors and bone cortex invasion [7,8]. Preoperative MRI is also considered the most useful method for evaluating mandibular invasion, an important issue in surgical treatment planning, which can be excluded due to the high negative predictive value (92.3%) of MRI in this setting [7]. The acquisition of ceCT as part of the combined PET/CT study, in addition to patient convenience, provides precise anatomic localization and delineation of the primary tumor when compared to non-enhanced PET/CT. In this context, iodinated contrast material has been shown to give only minor alterations in SUV measurement [47]. However, discordant results are reported with respect to nodal staging so that the clear diagnostic improvements of using PET/ceCT could not be demonstrated [39,40,48,49]. According to the ASCO guideline on “Diagnosis and management of squamous cell carcinoma of unknown primary in the head and neck”, ^18^F-FDG PET/CT is recommended in patients with metastatic cervical lymphadenectomy when the primary tumor is not evident on clinical examination and ceCT (strength of recommendation: strong) [16]. Indeed, less than 5% of patients with HNSCC present with cervical lymph node metastases, without clear evidence of the primary tumor at clinical examination, morphologic imaging, and panendoscopy [16]. Importantly, for this indication, PET/CT should be best performed prior to endoscopy, with the double benefit of reducing false-positive results and guiding biopsy [16,50]. In patients with metastatic cervical lymphadenectomy and unknown primary tumor at imaging, the detection rate of PET/CT is up to 42.5% [51,52,53]. According to a recent meta-analysis, the pooled detection rate was 40% (95% CI 31–49%) and the pooled false-positive rate was 9% (95% CI 5–13%) [17]. The most common sites of both true-positive and false-positive cases were the palatine tonsil and the base of the tongue, with false-positive results caused by high physiologic ^18^F-FDG activity at sites prone to inflammatory processes, such as the Waldeyer ring in the oropharynx [17,47]. In patients with an occult primary tumor, PET/CT results can impact therapeutic management, both by guiding surgical planning or directing individual changes in radiation-treatment volumes, which will cover only primary tumor sites instead of more extensive irradiation in case of an unknown primary [16]. Less frequently, a change in management in patients with an unknown primary is given by PET/CT through the detection of synchronous primary tumors or distant metastases (Figure 2).

### 3.2. Cervical Lymph Node Assessment

In patients with HNSCC, cervical LN involvement is one of the most important prognostic factors, as the presence of nodal metastases at initial diagnosis is the strongest predictor for nodal recurrence and the eventual development of distant metastases [54]. Survival is reduced by 50%, even with occult nodal involvement [55]. When LN metastases are documented, the disease is upstaged to stage III, even with T1/T2 primary lesions, thus requiring a multimodality therapeutic approach, i.e., surgery followed by adjuvant concurrent chemotherapy and radiotherapy or exclusive chemoradiotherapy [54]. HNSCC patients who are node negative can be spared from neck dissection, a surgical intervention that is associated with morbidity, particularly with damage to cranial nerves and shoulder dysfunction [56,57].

To detect nodal involvement in clinically negative patients (cN0), various imaging modalities can be used, including ultrasonography, ceCT, MRI, and ^18^F-FDG PET/CT, all showing suboptimal sensitivity and ranging between 52 and 71% [9]. In this setting, the role of sentinel node biopsy (SNB) in the clinically negative neck is more and more recognized. When compared with standard imaging modalities, SNB is the best-performing diagnostic tool for staging patients with cN0 HNSCC [58,59], with a negative predictive value of 96% [60] and a specificity of up to 99% [61]. Two large phase III randomized controlled trials confirmed that neck dissection can be safely avoided when SNB is negative, without affecting survival [62,63]. Despite these positive results, the widespread application of this procedure comes up against several limitations for its routine use in HN oncology, including the need for appropriate equipment and trained personnel; moreover, a multidisciplinary team including surgeons, nuclear medicine physicians, and pathologists is required [59]. 

**Figure 3:**
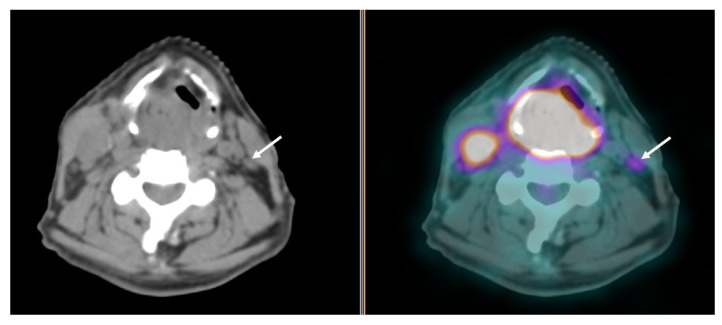
PET/CT images showing intense ^18^F-FDG uptake in a large hypopharyngeal cancer and an enlarged lymph node on the right cervical side; another sub-centimetric cervical lymph node with a mild increase in ^18^F-FDG uptake is seen contralaterally (arrows).

The literature data support the superiority of ^18^F-FDG PET/CT over ceCT and MRI in detecting LN involvement and defining its extent, with an overall sensitivity and specificity of 79–89% and 86%, respectively [56,64]. A clear advantage of functional imaging is that the size and structural criteria for LN involvement, which are applied for morphologic imaging are not required to visualize lesions by PET/CT, which detects metabolic changes in tumor cells. Due to its higher sensitivity in small nodal disease, ^18^F-FDG PET/CT allows for a better definition of the clinical target volume when primary exclusive chemoradiotherapy is indicated, with a higher dose on the involved nodal echelon. When the primary treatment is going to be surgery, nodal staging by ^18^F-FDG PET/CT allows for avoiding undertreatment and modifying the surgical planning, for example, by detecting positive LN(s) contralateral to the primary tumor in oral cavity SCC, thus leading to bilateral neck dissection (Figure 3), or occult retropharyngeal nodes in patients with oropharyngeal SCC, which would be missed by conventional neck dissection [56,65]. 

**Figure 4:**
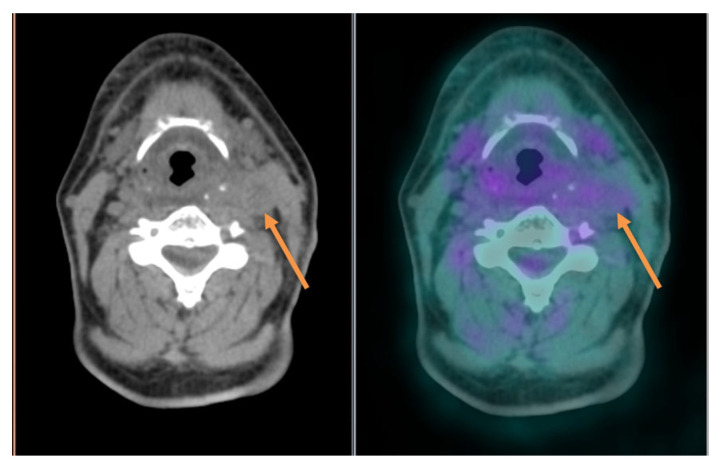
PET/CT images showing an enlarged and hypodense left cervical lymph node metastasis with no significant increase in ^18^F-FDG uptake due to necrotic changes (arrow).

Another situation when ^18^F-FDG PET/CT can be particularly useful for nodal staging is malignancies with an “intermediate” propensity to neck metastasis (regional relapse rate between 10 and 20% if the cN0 neck is not dissected) as SCC arising in the nasal vestibule. In these cases, the high sensitivity of ^18^F-FDG PET/CT would increase the reliability of nodal clinical staging. Moreover, when negative, it would allow a safer indication to observation, avoiding at the same time unnecessary neck dissections, which carry a risk of high morbidity due to the close anatomic relationship of the *marginalis mandibulae* nerve to the elective metastatic echelon (level IIB) [66,67]. However, both false-positive results in the case of inflammatory nodes and false-negative results in the case of necrotic or microscopic LNs are encountered. Indeed, necrotic LNs can escape from visualization at non-enhanced PET/CT as central necrosis, which is considered a reliable sign of metastatic LN and is clearly defined by ceCT, does not show FDG uptake [40,68] (Figure 4). 

Therefore, in selected cases, particularly human papillomavirus (HPV)-associated HNSCCs (most commonly oropharyngeal) or metastasis from an unknown primary in whom necrotic LNs are more frequently present, the acquisition of cePET/CT may be useful [40,68]. Also, microscopic LNs may be missed by PET/CT, [69] due to the finite spatial resolution of PET/CT scanners (2–4 mm with last-generation scanners) [26]. Indeed, in cN0 patients, the reported sensitivity of PET/CT in detecting LN involvement decreases to 50–71%, with significantly better results than ceCT or MRI in a few series only [56,63]. In the prospective, nonrandomized, multicenter trial of the American College of Radiology Imaging Network (ACRIN) 6685, in T2–T4 HNSCC, a high negative predictive value (about 87% for visual analysis and about 94% for SUV analysis) was shown, with a change in surgical planning in 22% of cases, thus suggesting that PET/CT results may help the clinician in deciding on the best therapeutic approach for cN0 HNSCC patients [70,71]. With a more widespread use of LAFOV systems, which are characterized by higher physical sensitivity and spatial resolution than standard scanners, a higher detection rate for micro-metastatic LNs in various tumors, including HNSCC, is expected [34,72].

### 3.3. Distant Metastasis Assessment

In HNSCC, the overall incidence of distant metastases at initial diagnosis is low, ranging from 2% to 18% [56]. Screening for distant metastases is critical in patients with advanced disease, particularly those with nodal involvement, in naso- and hypopharyngeal carcinomas, since the detection of distant metastasis prevents unnecessary aggressive surgery, and in recurrent disease. The most common sites are the lungs, bone, and liver. In this setting, according to the NCCN guidelines, ^18^F-FDG PET/CT is the preferred technique for patients with locoregionally advanced cancer (i.e., T3–T4 primary tumor and ≥N1 nodal staging) [2]. In this context, ^18^F-FDG PET/CT shows a high accuracy, particularly in detecting those bone metastases that can escape from CT detection. Exceptions are small lung lesions, which are better detected by a chest CT [2]. Also, brain metastases cannot be evaluated by ^18^F-FDG PET/CT due to the intense physiologic tracer uptake in the brain. However, they are a rare occurrence in HNSCC (less than 1%) [73]. In those cases, in whom these lesions are suspected (i.e., mucosal melanoma, neuroendocrine carcinoma, or adenocarcinoma), contrast-enhanced brain MRI should be performed [2].

### 3.4. Second Primary Tumor Assessment

**Figure 5:**
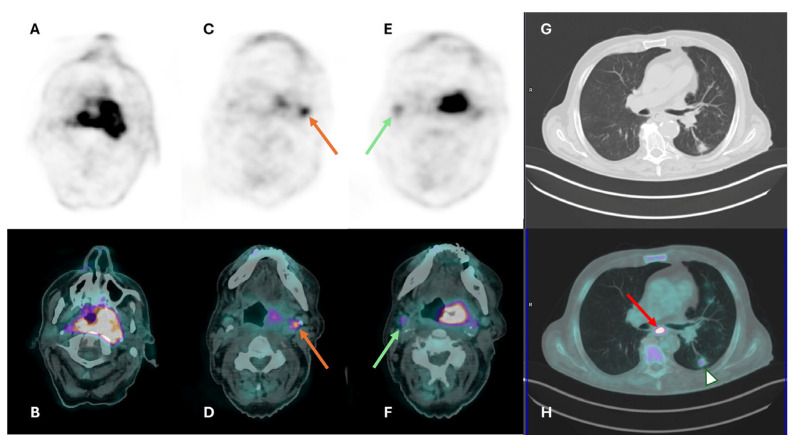
Axial ^18^F-FDG PET (**A**,**C**,**E**), CT (**G**), and fused PET/CT images (**B**,**D**,**F**) for staging in a patient with oropharyngeal carcinoma. Increased ^18^F-FDG uptake is seen in the primary tumor (**A**,**B**) with bilateral pharyngeal involvement (>on the left), as well as in bilateral cervical lymph nodes (yellow and green arrows in (**C**,**D**,**E**,**F**)). The focal ^18^F-FDG uptake in the esophagus (red arrow in (**H**)) was confirmed as a synchronous primary. Slight uptake by a pseudo-nodular left lung consolidation (white triangle in (**H**)) was due to inflammatory changes.

Second primary tumors, which can arise simultaneously (synchronous tumors) or subsequently (metachronous tumors), occur in 5–10% of HNSCC patients, mainly those who are smokers and HPV negative [74,75]. The most frequent sites of origin are the HN region, esophagus, and lungs (Figure 5); the less frequently reported sites are the thyroid, colon, breast, bile duct, and prostate [76]. The detection of a second primary tumor does impact patient treatment and management and requires a well-coordinated multidisciplinary approach. ^18^F-FDG PET/CT is an accurate method to detect second primary tumors, with a high negative predictive value of up to 100% [77]. 

As previously reported, to evaluate lung lesions, the standard workup of primary HNSCC includes a chest CT. However, differentiating second lung primaries from metastatic pulmonary nodules can be a challenge. Multiple lung nodules in the absence of advanced/recurrent nodal disease should be considered in the first instance as metastasis from another primary rather than from the known HNSCC. This is not an uncommon clinical scenario, one for which ^18^F-FDG PET/CT offers the incomparable advantage of detecting the second primary malignancy that, in these cases, is mostly located in the gastroenteric tract, namely the esophagus and colon. However, false-positive PET/CT findings are possible, such as inflammation and benign hyperplasia in the HN region or other sites that can concentrate ^18^F-FDG [10].

### 3.5. Prognostic Significance of Pre-Treatment PET/CT

Despite the recent advances in therapeutic strategies, the prognosis of patients with HNSCC remains poor, with a high recurrence rate (30–40%) [15]. Therefore, the identification of factors holding prognostic or predictive significance is of critical importance in guiding patient management. Patients with unfavorable prognostic factors may require more aggressive treatment, and patients with predictive biological markers may benefit from specific therapeutic strategies [78]. Traditionally recognized prognostic and/or predictive factors include tumor site and size, tumor grade and differentiation, depth of invasion, lympho-vascular and perineural invasion, and LN metastases [78]. More recently, biological markers, such as HPV detection in oropharyngeal SCC and Epstein–Barr virus detection in nasopharyngeal carcinomas, as well as specific genetic changes, have been applied (see below Section 3.6) [78]. In this context, the prognostic and predictive value of various ^18^F-FDG pre-treatment parameters in patients with HNSCC has been extensively investigated. Indeed, it is well known that ^18^F-FDG uptake into tumor cells is determined by many factors, such as the up-regulation of glucose transporters and hexokinase enzymes, neo-angiogenesis, and other factors, that reflect tumor aggressiveness and proliferative activity [79]. Among conventional imaging parameters, such as maximum, mean, peak standardized uptake values (SUV_max_, SUV_mean_, and SUV_peak_, respectively), metabolically active tumor volume (MTV), and total lesion glycolysis (TLG), the volumetric parameters MTV and TLG showed to be independent prognostic factors in most of the studies, with a higher prognostic value than SUV_max_ in terms of disease progression and overall survival [15]. In this scenario, an emerging role is played by radiomics, i.e., the extraction and analysis of various quantitative features from medical images including PET, which reflect tumor FDG distribution and, therefore, its heterogeneity. This topic is separately discussed in this review (see “Application of radiomics and machine learning”).

### 3.6. Proper Clinical Assessment and the Issue of HPV Involvement

Despite the histological homogeneity, HNSCCs are an extremely heterogeneous group of malignancies from a clinical point of view. Such heterogeneity must be taken into full account when interpreting PET/CT findings. Therefore, the more accurate the clinical staging, the more reliable will be the PET/CT report. In this context, the most relevant aspects are the primary site (for example, the retropharyngeal nodal metastasis as peculiar to the pharyngeal primaries, therefore, in these cases, an ^18^F-FDG uptake at that level should be always considered) and the virus-induced carcinogenesis. As previously mentioned, HPV-positive oropharyngeal SCC is characterized by a series of features that can deeply influence the interpretation of PET findings, in particular:

- the high rate of cystic/necrotic neck metastasis with a typical low ^18^F-FDG uptake [68];
- the low rate of second primary tumors (mainly lung and esophagus) [75];
- the slow response with a longer persistence of increased SUV, particularly in neck nodes, even in case of complete response after chemoradiotherapy [14].

Therefore, the correct definition of HPV-driven carcinogenesis as part of the work-up is of paramount importance, and the possibility of false-positive findings at sole p16-immunohistochemistry should not be underestimated [80,81]. In populations with a rate of HPV-driven SCC in the oropharynx below 40%, p16 overexpression should be integrated with nucleic acid detection to confirm HPV-driven carcinogenesis and draw proper clinical considerations [82,83]. 

## 4. Radiotherapy Planning

PET/CT imaging, particularly with ^18^F-FDG, has emerged as a cornerstone in enhancing radiation-treatment planning in locally advanced HN cancers by defining patient selection and the goal of radiation treatment, as well as through accurate delineation of target volumes and treatment adaptation, facilitating intended management and dose escalation, and potentially reducing treatment-related toxicity (Table 1) [84].

As described above, a well-known distinctive advantage of ^18^F-FDG PET/CT is its ability to detect hidden primary tumors in patients with metastatic cervical LNs from unknown primaries. This reduces the radiotherapy target volume, minimizing treatment side effects [16,17]. Moreover, technological innovations, with the development of PET/CT devices with higher physical sensitivity and spatial resolution, can lead to increased detection rates of small nodal metastases, altering tumor load in the target volumes and impacting radiotherapy dosing with the inclusion of low-volume disease in high-dose volumes [85]. In clinical routine, visual analysis with manual contouring is the most used segmentation method, even though its use is hampered by display windowing and the subjective nature of the analysis. Other methods include the use of quantitative parameters derived from ^18^F-FDG PET/CT, such as SUV_max_, MTV, and TLG [85,86]. These parameters can be considered in the choice of personalized doses and volumes in radiotherapy treatment planning, as demonstrated in “dose painting” by numbers studies, in which the dose delivered to each voxel of the target is based on the signal intensity of that voxel on the PET image [87]. PET/CT can potentially modify initial radiotherapy planning in a considerable fraction of patients due to its enhanced accuracy in delineating gross tumor volumes (GTV), sometimes offering a more defined GTV compared to those obtained from ceCT or MRI [88,89,90,91]. However, the primary limitation of this approach arises from the absence of standardized methods for segmenting functional volumes, significantly impacting the resulting GTV and shape [88,89]. International consortia have been established to define shared contouring guidelines, identifying and distinguishing—by the co-registration of PET/CT images with CT images for radiation-treatment planning—a high-risk volume, an intermediate-risk volume, and a low-risk volume with different prescription doses [88,89]. Furthermore, ^18^F-FDG PET/CT may not reliably detect small superficial tumor deposits or nodal micrometastases, emphasizing the importance of clinical assessment and the need for further improvements in imaging technologies for radiation-treatment planning in HNSCC [92]. 

Loco-regional failure is a common event in locally advanced HN cancer, with up to 50% of patients having a poor prognosis [93]. Most of the loco-regional failures in patients undergoing intensity-modulated radiation therapy (IMRT) occur within areas receiving the highest prescribed radiation dose [94]. While an increased radiation dosage can enhance local control due to a dose–response relationship, intensified treatment targeting the entire GTV may increase toxicity, necessitating cautious consideration [95]. Tumor sub-volumes with increased metabolism and hypoxia often exhibit increased radio resistance, making them more resistant to standard treatment approaches, as documented in several studies where PET/CT with specific hypoxia tracers was used to personalize radiotherapy doses [96,97,98]. Boosting radiotherapy to specific tumor sub-volumes, such as hypoxic or resistant areas, may lead to improved local control and patient outcomes. The FiGaRO trial evaluated the safety and feasibility of using ^18^F-FDG PET/CT-based dose painting with intensity-modulated radiotherapy to administer a boost to the ^18^F-FDG-avid primary tumor in locally advanced high- and intermediate-risk oropharyngeal cancers, showing comparable late toxicity rates to standard-dose chemo-IMRT and suggesting improved 3-year survival rates for high-risk patients [99]. ^18^F-FDG PET-guided dose escalation has been investigated in multiple phase 1 trials and a recently published phase 3 trial [100]. The latter study, performed in a mixed population with locally advanced HNSCC showed no significant improvement in locoregional control, progression-free survival, or overall survival. A comparable two-year toxicity rate with respect to conventional treatment was observed. However, the sub-group analysis of patients with oropharyngeal and stage N0–1 cancer treated with ^18^F-FDG PET-guided dose redistribution showed improved locoregional control compared to the control group. These preliminary results should be further investigated by analyzing treatment efficacy and outcome after ^18^F-FDG PET-guided dose-escalation according to different tumor sites and stages [100].

Tracers like ^18^F-fluoromisonidazole (^18^F-FMISO) and ^18^F-fluoroazomycin-arabinofluranoside (^18^F-FAZA) are under investigation for their ability to provide quantitative evaluations of tissue hypoxia [101]. This topic is separately discussed in this review (see “PET radiopharmaceuticals other than ^18^F-FDG”).

PET/CT can play a central role in the complex topic of replanning in radiotherapy. Indeed, radiotherapy adaptation through replanning is a useful procedure for correcting patient anatomical changes related to possible weight loss during therapy, or it could be necessary when target volumes need to be redefined due to a relevant clinical response of the disease. In these cases, PET/CT imaging during treatment can lead to personalized care by considering the clinical behavior of the tumor. The mid-treatment metabolic imaging can guide strategies of dose and volume modulation to propose treatment personalization through a new radiotherapy plan that considers the biological response of the tumor; the reduction of GTV revealed by ^18^F-FDG PET/CT at mid-treatment evaluation potentially leads to changes in the radiation dose with decreased toxicity and improved local control [102]. Moreover, mid-treatment assessments using ^18^F-FDG PET/CT scans offer valuable insights into treatment-response prediction, shaping the blueprint for adaptive clinical trials [103]. A recent randomized phase II trial demonstrated the efficacy of a PET-based adapted dose escalation approach in enhancing local control of HN cancer over traditional IMRT, supporting the need for further exploration in larger phase III trials [104].

## 5. Treatment-Response Assessment

**Figure 6:**
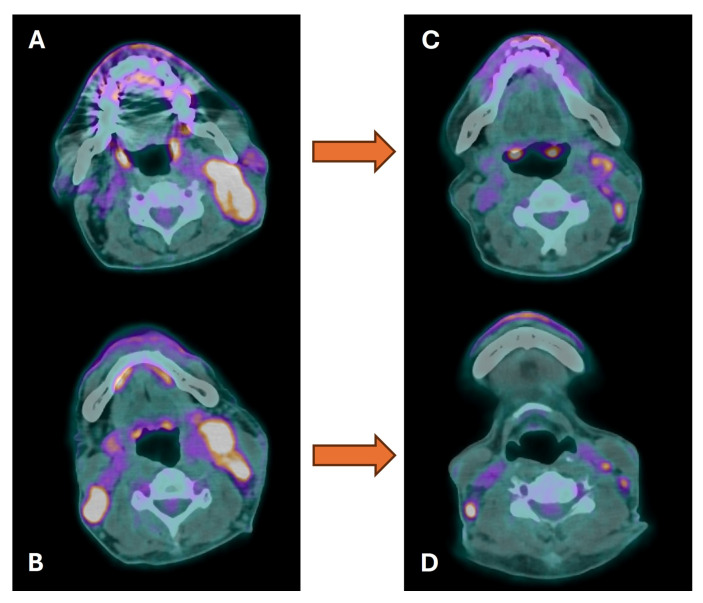
Baseline (**A**,**B**) and post-chemotherapy (**C**,**D**) ^18^F-FDG PET/CT in a patient with bilateral cervical lymph node metastases from nasopharyngeal carcinoma. Marked reduction in entity and extent of ^18^F-FDG, as well as in size, was observed in all the involved lymph nodes, with a persistent inhomogeneous uptake and no evidence of new active lesions (partial response).

Current international guidelines support the use of imaging 3–6 months after the primary treatment in patients with locally advanced HN cancer to assess treatment response, identify residual tumors, and have a baseline post-treatment imaging examination [1,2,105]. Because of its high negative predictive value (94–97%) with optimal performance approximately 3 months after the end of the treatment, ^18^F-FDG PET/CT can reduce the number of unnecessary invasive procedures or therapeutic interventions [1,18,106,107,108]. A prospective, randomized, controlled trial in more than 550 patients with HNSCC and N2 or N3 disease found that ^18^F-FDG PET/CT after primary chemoradiotherapy was associated with a reduction in neck dissections, fewer surgical complications, and adverse effects and, finally, with lower treatment costs [14]. Currently, neck dissection is not recommended in cases of negative ^18^F-FDG PET/CT and normal-size LNs 3 months after chemoradiotherapy [1]. ^18^F-FDG PET/CT performed 3–6 months after the primary treatment is also correlated with overall and disease-free survival [105,109,110]. Early ^18^F-FDG PET/CT, 3 months after the end of radiotherapy, is associated with significant false-positive rates and is not recommended in the absence of signs of recurrence or progression [2]. Early after chemoradiotherapy, the positive predictive value and specificity are lower, possibly due to the increased vascularity, edema, and inflammatory changes related to treatment. However, diffuse ^18^F-FDG uptake at the primary site and in the neck is more consistent with inflammation related to post-therapy changes, whereas intense and focal ^18^F-FDG uptake is more likely related to residual disease (Figure 6). Several criteria have been proposed to evaluate treatment response to ^18^F-FDG PET/CT in patients with HN cancer, including the Hopkins and the Head and Neck Imaging Reporting and Data System criteria (NI-RADS) [111,112] (Table 2). According to the Hopkins criteria, the lesions are classified according to a five-point scale relative to physiologic ^18^F-FDG avidity in reference structures, the internal jugular vein, and the liver. In NI-RADS, the ^18^F-FDG PET/CT results are combined with anatomical imaging findings, and the scores range from zero to four. However, NI-RADS demonstrated many indeterminate cases, most likely due to the absence of a reference standard for ^18^F-FDG uptake, which may lead to poor inter-reader reproducibility [113]. Treatment response can be assessed qualitatively or semi-quantitatively by the calculation of several PET metrics (e.g., SUV, MTV, and TLG). Both methods can be used to interpret PET scans with a reliable degree of accuracy [87,107].

Quantitative evaluation allows a more objective comparison of metabolic activity in the same lesion over time and is commonly used in clinical trials to predict outcomes. Quantitative analysis has typically been used to evaluate the ability of ^18^F-FDG PET/CT to predict early response during chemoradiotherapy or radiotherapy to intensify treatment in case of non-response or to reduce the intensity of the remaining therapy in patients who achieve a complete response [104,114]. At interim PET, reduction of SUV_max_ or an MTV higher than 50% in the primary tumor was associated with higher 2-year overall survival and locoregional control [103,115], and an SUV_max_ reduction ratio < 0.64 resulted in lower 2-year overall survival and disease-free survival [116]. However, larger sample-sized studies and external validation of metabolic parameters are needed to implement adaptative treatment guided by functional imaging.

**Table 2:** Standardized reporting systems for ^18^F-FDG PET/CT: Hopkins criteria and Neck Imaging Reporting and Data System [111,112].

Criteria		
**HOPKINS**	**^18^** **F-FDG uptake pattern at the primary site and nodes**	**Response category**
**1**	focal uptake less than IJV	Complete metabolic response
**2**	focal uptake, greater than IJV but less than liver	Likely complete metabolic response
**3**	diffuse uptake greater than IJV or liver	Likely inflammatory changes
**4**	focal uptake greater than liver	Likely residual tumour
**5**	focal and intense uptake	Residual disease
**NI-RADS**	**Primary site response**	**Management recommendations**
**0**	Incomplete and baseline imaging not available	Assign score after availability of prior scan
**1**	No evidence of recurrence	Routine surveillance, CECT
**2**	Questionable recurrence: a. Superficial abnormality (skin, mucosal surface, etc.) b. Deep abnormality < 1 cm with mild/intermediate ^18^F-FDG c. Deep abnormality > 1 cm with mild/intermediate ^18^F-FDG	Direct visual inspection Short interval follow-up PET/CECT Short interval follow-up or biopsy if clinically indicated
**3**	High suspicion of recurrence: new discrete nodule or mass, ^18^F-FDG avid	Biopsy if clinically needed
**4**	Known recurrence, biopsy proven	Clinical management
	**Node response**	
**1**	No evidence of nodal disease recurrence	Routine surveillance
**2**	Questionable nodal recurrence or residual nodal disease: a. <1.5 cm with mild/intermediate ^18^F-FDG b. >1.5 cm with mild/intermediate ^18^F-FDG	Surveillance Biopsy or short-interval follow-up
**3**	High suspicion of recurrence (new, enlarging, FDG avid)	Biopsy if clinically needed
**4**	Known recurrence, biopsy proven	Clinical management

IJV: internal jugular vein; NI-RADS: Neck Imaging Reporting and Data System.^18^F-FDG PET/CT is effective for detecting early asymptomatic lesions generally occurring at distant sites [117]. Whether earlier detection leads to improved disease-specific survival is not established. In patients with negative ^18^F-FDG PET/CT at 3 months post-treatment, subsequent surveillance should be tailored according to tumor type, stage, prognostic factors, symptoms, and physical exam changes.

## 6. Long-Term Follow-Up (≥6 Months to 5 Years Post-Treatment)

Approximately 50% of patients with locally advanced HNSCC relapse after primary treatment with distant metastases and/or local or regional disease in the first 2 years [118]. Post-treatment imaging is recommended if symptoms appear, if there are abnormalities on clinical examination, or if detection of the tumor may be difficult by clinical examination or direct inspection only [2]. There are no consensus guidelines on the frequency and modality of routine post-treatment imaging in asymptomatic patients. The literature data show that ^18^F-FDG PET/CT performed 1 year after treatment can reveal recurrent or second primary cancers in approximately 10% of patients, and 2-years ^18^F-FDG reveals these findings in approximately 5% of treated patients [105].

## 7. Cost-Effectiveness Analysis

As previously described, current guidelines support the adoption of a PET/CT-guided approach into routine clinical practice for HN cancer patients treated with chemoradiotherapy, i.e., avoidance of neck dissection in patients with negative PET/CT performed at 3 months after completion of chemoradiotherapy [2]. This approach, in addition to showing clinical utility in terms of outcome, also has economic implications. Initial non-randomized studies—one performed according to Australian health care [119] and three according to United States health care [120,121,122]—found that ^18^F-FDG PET/CT is a cost-effective alternative to neck dissection. These observations were confirmed by the large United Kingdom clinical trial PET-Neck, a phase III prospective, randomized, controlled trial performed on patients with loco-regionally advanced HN cancer (oropharyngeal, laryngeal, oral, hypopharyngeal, or occult, with stage N2 or N3 and M0 disease) who received chemoradiotherapy for primary treatment. This study demonstrated that PET/CT-guided surveillance, when compared to neck dissection, was cost-effective over a short-term period (at least 2 years follow-up post-randomization), resulting in far fewer operations (about 80% of patients were spared from surgery) and in saving GBP 1492 (approximately USD 2190) per person [14]. The quality of life was similar in the two groups. Comparable results were subsequently reported by extrapolating the data over a long-term period (additional follow-up up to 5 years) [123].

## 8. PET Radiopharmaceuticals Other Than ^18^F-FDG

In the last years, more tumor-targeted PET radiopharmaceuticals, which reflect biologic characteristics of HN tumors have been proposed, either in specific clinical settings or in different histotypes (Table 3) [19]. These radiopharmaceuticals may overcome the most relevant drawbacks of ^18^F-FDG, like non-specific uptake due to inflammation, which could hamper its diagnostic worth in terms of specificity and positive predictive value (especially after radiotherapy), or high physiological uptake by anatomical structures usually harboring HN cancer, which leads to a reduced tumor-to-background ratio. This is of clinical significance in patients with an unknown primary neoplasm or for detecting skull-base invasion or brain/skull metastases. ^18^F-FDG also suffers from very low uptake in some well-differentiated histotypes, including their metastases and, therefore, is not suitable for staging, restaging, and treatment efficacy evaluation in these patients.

Hypoxia is a common event and the main cause of local failure after radiotherapy in HN cancer [124,125]. Hypoxic volumes measured using ^18^F-FMISO and ^18^F-FAZA PET/CT at staging are more prone to loco-regional recurrence regardless of primary tumor grading, and also in HPV-positive patients. Non-invasive detection of hypoxia is useful for tumor “dose painting”, that is, dose escalation to the PET-depicted hypoxic volumes, without an increased risk of damage to neighboring critical structures [20,101,126,127,128] and possibly carrying a lower risk of loco-regional recurrence in comparison with standard protocols [20]. Indeed, hypoxic sub-volumes may be unevenly distributed throughout the gross tumor volume visible on a CT or an MRI. Therefore, the extent of the disease amenable to receive a dose boost may be much lower, and with a completely different distribution, than the anatomical lesion. This makes it possible to spare sensitive structures (e.g., nerves, vessels…) anatomically enclosed by the tissue. However, variations in tumor size and geographical distribution of hypoxic volumes during radiotherapy, coupled with radiation-induced inflammation or edema, and re-oxygenation phenomena, may reduce the effectiveness of dose escalation protocols on local and/or distant disease control [101,129,130]. In this view, performing serial PET/CT scans during radiotherapy may identify patients with worse local control who exhibit persistent hypoxia just 2 weeks after radiotherapy started [131]; also, tumor sub-volumes at higher risk of recurrence are identified, which are persistently hypoxic across baseline and during-treatment PET/CT imaging [129]. Interestingly, in most HN cancer lesions, hypoxic volumes depicted using ^18^F-FMISO are not correlated spatially to the areas with the highest ^18^F-FDG uptake. Therefore, ^18^F-FDG should not be used as a surrogate to predict hypoxia [132]. 

A clear advantage of fibroblast-activating protein inhibitors (FAPi) over ^18^F-FDG is their high tumor-to-background contrast that allows an exceptionally clear tumor delimitation in districts with high ^18^F-FDG background uptake (e.g., brain, liver, spleen, bowel, and tonsils) [133]. As reported in a recent systematic review and meta-analysis [134], FAPi PET/CT is very useful for detecting unknown primary HN tumors in patients with cervical LN metastases, thanks to higher uptake than surrounding healthy tissue, and is better than ^18^F-FDG in assessing skull-base invasion due to negligible brain uptake (superior performance than MRI in patients with nasopharyngeal carcinoma) [135]. Moreover, FAPi PET/CT is helpful for detecting cervical LN metastases from HN cancer, with acceptable sensitivity (80–90%, false-negative findings in lesions < 5 mm) and higher specificity than ^18^F-FDG (93.3% vs. 81.3%) due to less inflammatory false-positive nodes [21,136]. FAPi PET/CT is superior to ^18^F-FDG in detecting distant metastases, especially in the bone and brain [133,135]. 

Methionine is an essential amino acid incorporated into proteins; when labeled with carbon-11 (C-MET), it may be used as a radiopharmaceutical for detecting protein synthesis in malignant and benign conditions. Its ability to detect response to conventional radiotherapy and carbon-ion therapy in adenocarcinomas, adenoid cystic carcinomas, and even more uncommon histologies (e.g., mucosal melanoma) has been confirmed in the last 30 years [22,137,138]. In patients undergoing radiotherapy or carbon-ion therapy, higher residual C-MET uptake at post-treatment PET/CT predicts an increased risk of local recurrence and, in patients with mucosal melanoma, also of distant metastases [137,138,139]. Moreover, patients with suspected HN cancer recurrence may benefit from the higher specificity of C-MET (76% vs. 56%) after equivocal ^18^F-FDG PET/CT, thus reducing the need for targeted biopsies [23].

Fluorine-18 labeled amino acid-based radiopharmaceuticals have been proposed, particularly, O-2-fluoro-^18^(F)-ethyl-L-tyrosine (^18^F-FET), which enters tumoral cells using an over-expressed trans-membrane transport system. However, since its first reported use in 2006 [140], it has demonstrated lower sensitivity than ^18^F-FDG and is, therefore, not suitable as an alternative to ^18^F-FDG in the evaluation of HN cancer patients [141]. Also, ^18^F-FDG detects more primary tumors than ^18^F-FET, as well as more distant metastases and second primitive lesions than ^18^F-FET. On the other hand, ^18^F-FET has higher specificity, especially in lymph nodes, due to negligible uptake in inflammation sites [140,141,142]. 

A labeled modified thymidine-derived nucleoside, 3′-deoxy-3′-^18^F-fluorothymidine (^18^F-FLT), may be used as an in vivo marker of cell proliferation. The ^18^F-FLT uptake intensity within the lesion is associated with a worse prognosis and, therefore, may impact on patients’ treatment [124]. In agreement with this finding, HPV-negative patients exhibit higher SUV_max_ on ^18^F-FLT PET/CT and have worse prognosis [143]. Moreover, baseline ^18^F-FLT uptake and metabolic volume predict local and distant disease control after chemoradiotherapy [144] and are better predictors of overall survival than ^18^F-FDG [144] thanks to the negligible inflammatory uptake [145]. ^18^F-FLT PET/CT may also be used for monitoring the early response to radiotherapy in HN cancer, allowing dose escalation in sites of residual ^18^F-FLT uptake after the first treatment, with the achievement of complete tumor regression [24]. 

PET/CT imaging specifically designed for investigating HN neuroendocrine tumors includes somatostatin analogs against over-expressed somatostatin receptors (SSTR), like ^68^Ga-DOTATOC, ^68^Ga-DOTANOC, and ^68^Ga-DOTATATE, or norepinephrine precursors such as ^18^F-DOPA. Particularly, PET/CT with SSTR ligands exhibits higher accuracy in detecting HN paragangliomas and their metastases over morphological imaging, particularly very small lesions [25,146,147,148]. Loss of SSTR expression because of tissue de-differentiation explains most false-negative findings and is associated with a worse prognosis; these lesions are amenable to detection using ^18^F-FDG. The NCCN guidelines recommend PET/CT with SSTR ligands to exclude distant metastases in patients with HN paraganglioma, while EANM guidelines consider PET/CT with SSTR ligands as the first-choice functional imaging in sporadic and hereditary HN paragangliomas and, as the second choice, PET/CT using ^18^F-DOPA or ^18^F-FDG [149,150,151].

## 9. The Role of PET/MRI

PET/MRI scanners combine the high accuracy of MRI in the evaluation of the primary tumor (local extent, perfusion, and structural characteristics) with the high sensitivity of PET for distant metastases, nevertheless with lower radiation exposure (only from the PET component). In the evaluation of a primary tumor, ^18^F-FDG PET/MRI using gadolinium-enhanced T1-weighted sequence has a clear advantage over PET/CT in detecting the infiltration of neighboring structures and peri-neural spread [152,153,154,155,156], especially in pharyngeal and oral lesions, whereas MRI is less prone to artifacts from dental hardware than PET/CT [157,158]. Moreover, gadolinium contrast-enhanced PET/MRI has been demonstrated to perform better than cePET/CT in early detection of peri-neural spread [157], which is a well-known negative prognostic factor for disease progression. PET/MRI also has the ability to better distinguish tumor uptake from sites of physiological accumulation compared to PET/CT, due to a clearer definition of anatomic structures [159,160], especially when PET and MRI are acquired simultaneously (fewer misregistration artifacts) [161]. 

The diagnostic advantage of PET/MRI over PET/CT or MRI alone in the nodal staging of patients with nasopharyngeal and hypopharyngeal cancer is not fully established [162,163,164], despite fewer false-positive findings in assessing N2–3 status being reported using PET/MRI over the standard diagnostic work-up [165]. In some papers, including mixed HN cancer histology, PET/MRI results are as accurate as cePET/CT in N-staging [166,167,168,169,170,171] and can be considered a valid tool in patients with allergy, renal failure, or other contraindications to iodinated contrast medium [157]. PET/MRI performs well in detecting distant metastases (mostly in the lungs), possible second primary tumors, or an otherwise occult primary lesion in the head and neck. Particularly, the very high NPV of PET in lung nodules overcomes the well-known limitations of MRI in studying lung parenchyma, therefore excluding lung metastases with high reliability. In addition, new MRI sequences designed for studying pulmonary parenchyma may detect nodules even smaller than 4 mm [172,173,174]. ^18^F-FDG PET/MRI is more sensitive and specific than PET/CT in patients with cervical unknown primary [175,176]. In this setting, there is interest in applying PET/MRI for the delineation of the radiotherapy field by using PET-derived (e.g., SUV_max_ % threshold or SUV_max_ absolute value) and MRI-derived (GTV from contrast-enhanced T1 and T2 sequences, or from T1-weighted VIBE or Dixon sequences) volumes, with better soft-tissue contrast and lower radiation exposure [177,178]. However, despite technological advances, PET/MRI still overestimates, or sometimes partially misses, pathological GTV, especially in patients with smaller lesions [179]. PET/MRI is useful for the detection of tumor persistence or recurrence after treatment thanks to the high soft-tissue contrast of MRI, which discriminates pathological tissue from post-treatment alterations, and the high NPV of PET. Moreover, PET/MRI exhibits a significantly higher diagnostic accuracy than MRI alone in detecting the recurrence of low-FDG uptake malignancies, such as adenoid cystic carcinoma, especially in detecting peri-neural spread and metastases in small lymph nodes which were deemed reactive on an MRI [180]. 

Although “functional” MRI techniques (diffusion weighted, dynamic contrast enhanced, or spectroscopy) may provide additional information for tissue characterization, response prediction, and outcome, the real usefulness in the clinical practice is questionable, since PET already provides much of this information [181,182]; moreover, spectroscopy is time-consuming and needs a specialized team [183]. The main drawback of widely using PET/MRI in clinical practice is the need for an alternative technique for PET photon attenuation correction (MRI is not a measure of tissue density unlike CT). Therefore, an attenuation map should be obtained from MRI sequences (as T1-weighted DIXON) to segment the patient’s tissues into air, lungs, soft tissue, and fat. Then, an anatomic atlas is applied to add bone segmentation. Moreover, an MRI is time-consuming, and at least attenuation-correction MRI sequences should be acquired simultaneously with PET images to avoid exceedingly long-lasting examinations. Additional diagnostic MRI sequences of the head and neck should be performed at the end of PET acquisition, therefore taking more time away, and it is difficult to apply them systematically when facing a busy patient list. In addition, performing PET/MRI is more expensive than PET/CT, and a two-hand reporting (nuclear medicine physician and radiologist) is needed for all patients undergoing PET/MRI [165].

## 10. Application of Radiomics and Machine Learning

Although semi-quantitative parameters consider the volumetric extent of active areas, like MTV or TLG, and adequately mirror the tumor heterogeneity, which is a well-known negative prognostic factor in HN cancer, their effectiveness and reproducibility for predicting a clinical outcome may be hampered by the need for an “a priori” established SUV threshold for lesion contour [184]. Radiomics is an advanced “data mining” technique able to extract information from high-resolution images by measuring the transitions of intensity between adjacent voxel values and their mutual relationships not visible to the naked eye. By this, radiomics detect a subtle heterogeneity in uptake distribution across the lesion, exploring non-invasively its complex structure and biological characteristics in detail [185]. Moreover, the ever-increasing computational performances achieved in recent years have led to the development of informatic systems able to imitate human intelligence, so that they may learn from provided data, build up artificial neural networks, and perform their tasks without further man-initiated instructions. This is how machine-learning models work, and, when applied to radiomics, they can analyze quickly the usually high number of extracted features and find possible relationships among them or specific patterns for prognosis prediction [36,186,187]. 

Recent studies and meta-analyses have demonstrated a reasonable performance for outcome models obtained from radiomics analysis in HNC [19], confirming that more homogeneous tumors have a better prognosis [188,189,190,191] and that the combination of radiomics and clinical information excellently predicts PFS and OS [192]. Moreover, in patients with ongoing radiotherapy, baseline to post-treatment differences in PET/CT radiomics may predict PFS and OS, irrespective of clinical parameters and T and N stage [193]. Radiomics analysis also performs better than clinicopathological factors in predicting cervical lymph node metastases [194]. Several studies have explored the ability of ^18^F-FDG PET/CT radiomics-based machine-learning analysis for predicting treatment outcomes in HN cancer [190,195,196,197,198,199,200,201,202], reporting a good performance of the radiomic features (alone or combined with genomic data and T and N stage) in predicting loco-regional progression, PFS, 3-year OS, or recurrence-free survival [196,203,204], with higher accuracy than SUV and TLG in distinguishing local recurrence from post-treatment inflammation and predicting local failure [196,205,206]. Deep learning applied to PET/CT in HN cancer has demonstrated high diagnostic accuracy, sensitivity, and PPV in differentiating treatment control and failure, better reflecting the disease-free survival rate than T stage, clinical stage, SUV_max_, SUV_mean_, MTV, and TLG [37,207]. In addition, in the view of delivering more dose to the radio-resistant part of the tumor (“dose painting”), the feasibility of applying radiomics analysis to ^18^F-FDG PET/CT scans at baseline and during/after chemo-radiation in HN cancer patients has been explored, finding it is clinically suitable to distinguish radio-resistant and radio-sensitive volumes within the same lesion [208]. The main limitations to the widespread application of radiomics and machine learning in evaluating patients with HN cancer rely on the lack of standardized acquisition and reconstruction parameters, which may vary when using different scanners and, therefore, potentially reducing their clinical reliability and lowering intra- and inter-institutional reproducibility [209]. Moreover, the use of non-standardized segmentation techniques for the lesions and organs at risk may interfere with the subsequent features extraction and the selection phase (this is of particular relevance in patients amenable to radiotherapy) [210]. Finally, specific training for the clinical personnel is required to ensure adequate management and interpretation of the results [211]. 

## 11. Conclusions

^18^F-FDG PET/CT is the standard of care for patients with advanced stage head–neck squamous cell carcinoma, having a significant impact on patient management and outcome. Current international evidence-based guidelines support its use in various clinical settings of this tumor, ranging from initial staging and radiotherapy planning to treatment-response assessment and detection of recurrence, due to its higher sensitivity over clinical examination and conventional morphologic imaging. The use of standardized methods for reporting therapy response and the excellent negative predictive value of post-therapy ^18^F-FDG PET/CT further contribute to the significant impact of this imaging modality on clinical practice. A wider diffusion of PET/MRI and the technological innovations by LAFOV PET/CT systems, as well as the use of PET radiopharmaceuticals other than ^18^F-FDG and the implementation of radiomics and machine learning, will answer specific clinical needs, further improving patient management and outcome.

## Figures and Tables

**Table 1:** Role of PET/CT for radiotherapy planning.

PET/CT and Radiotherapy Planning	Clinical Examples
Patient selection and intended management	Treatment (local disease) versus non-treatment (distant metastases)
Goal of treatment	From curative to palliative and vice versa
Selection and delineation of GTV	- Detection of occult primary tumor (see text) - Tumor extension not defined on CT or MRI (see Figure 3)
Dose painting based on biological tumor features	Dose escalation to ^18^F-FDG avid or hypoxic sub-volumes
Adaptive radiotherapy	Escalation or de-escalation during treatment

GTV: gross tumor volume; CT: computed tomography; MRI: magnetic resonance imaging.

**Table 3:** PET radiopharmaceuticals other than ^18^F-FDG.

Radiopharmaceutical	Molecular Target	Main Indications	Clinical Application
^18^F-FMISO ^18^F-FAZA ^18^F-EF5 ^18^F-FETNIM ^18^F-HX4 ^64^Cu-ATSM	Hypoxia	Staging Response evaluation Adaptive Radiotherapy	Experimental
^68^Ga-FAPi ^18^F-FAPi Al^18^F-NOTA-FAPi	Fibroblast Activating Protein (FAP)	Staging Unknown primary	Experimental
^11^C-MET	Protein synthesis	Adaptive radiotherapy Response evaluation	Clinical
^18^F-FET	Protein synthesis	Staging–Restaging	Experimental
^18^F-FLT	Cell proliferation	Staging–Restaging Response evaluation Adaptive radiotherapy	Experimental
^68^Ga-DOTATOC ^68^Ga-DOTANOC ^68^Ga-DOTATATE	SSTR-expression	Staging–Restaging Response evaluation Targeted therapy	Clinical
^18^F-DOPA	Neurotransmitter transportation	Staging–Restaging	Clinical
